# The Diagnostic Performance of the Cellavision DC-1 Digital Morphology Analyser on Leukaemia Samples

**DOI:** 10.3390/diagnostics15162029

**Published:** 2025-08-13

**Authors:** Annabel Kowald, Chun Ho Fung, Jane Moon, Sapha Shibeeb

**Affiliations:** School of Health and Biomedical Sciences, RMIT University, Melbourne, VIC 3000, Australia; annarkowald@gmail.com (A.K.); chunho.evanfung@hotmail.com (C.H.F.); sapha.shibeeb@rmit.edu.au (S.S.)

**Keywords:** Leukaemia, haematological disease, AI-assisted diagnosis

## Abstract

**Background/Objectives:** Digital morphology analysers have been developed to overcome the limitations of manual microscopy. This study aimed to evaluate the performance of the DC-1 on leukaemia samples, determining if it is a suitable for the identification of leukaemia in low-throughput or remote laboratories. To the best of our knowledge, there is no current published literature evaluating the performance of the DC-1 with leukaemia samples. **Methods:** This study utilised 88 leukaemia peripheral blood smears donated from various anonymous hospitals and medical laboratories in collaboration with RMIT university. DC-1 pre-classification was compared with post-classification using Cohen’s kappa, sensitivity, and specificity calculations. Pre- and post-classification was compared with manual microscopy using Passing–Bablok regression, Pearson’s r correlation, and Bland–Altman analysis. **Results:** DC-1 pre-classification results showed a moderate agreement with post-classification (k = 0.52), a very high specificity for most leukocytes (>94%) and variable sensitivity (21–86%). Pre- and post-classification displayed a higher accuracy and correlation with manual results for segmented neutrophils and lymphocytes, compared to other leukocyte classes. Additionally, there was an improvement in the post-classification of immature granulocytes, band neutrophils, and blast cells compared to pre-classification. **Conclusions:** The results indicate that the DC-1 displayed a better performance for the classification of segmented neutrophils and lymphocytes compared to other cell classes, indicating that the DC-1 is more acceptable for use in infection or normal samples, as opposed to leukaemia. The gold standard therefore remains with the morphologist who can distinguish leukaemia samples.

## 1. Introduction

The morphological evaluation using peripheral blood smears is a fundamental process in medical laboratories and is essential for the diagnosis of haematological diseases [1]. Despite its routine use, there are some limitations associated with manual microscopy, with the most frequent concerns being turnaround time, inter- and intra-observer variation, and the need for an adequate level of morphology training and experience [2]. In recent decades, digital cell morphology analysers have been developed and implemented with the objective of improving on the limitations associated with manual microscopy [3]. There are various digital morphology analyser models available that differ in their image analysis software and peripheral blood smear scanning capacity. Automated pre-classification of cells based on textural, colour, and geometric features is made possible through the utilisation of a digital camera and analysis software, coupled with artificial intelligence [4]. Machine learning from statistical algorithms aids in morphological cell classification by neural networks [5]. Neural networks that have been used by digital morphology analysers to classify cells include convolutional neural networks (CNNs) and artificial neural networks (ANNs) [4,5]. Findings from previous studies concerning digital morphology analysers mostly correlate and display a satisfactory to high accuracy for the detection of neutrophils and lymphocytes, as well as correlation with manual microscopy for normal blood smears [2,6,7,8]. However, there appears to be variation between analyser models and a decreased performance for the classification of eosinophils, basophils, abnormal lymphocytes, immature granulocytes, and blast cells [4,6,9,10,11,12]. Samples with an abnormally increased or decreased leukocyte count also produced less accurate results, with the quality of the staining and smear also affecting the ability of the analyser to complete scanning and classify leukocytes accurately [4,6,13].

The Cellavision DC-1 digital morphology analyser is a compact, low-throughput, semi-automated analyser that is designed to be used in laboratories with a small to medium workload due to its scanning capacity of one slide at a time [1,14,15]. This analyser may be particularly beneficial for remote or low-volume laboratories that might face a shortage of medical scientists with adequate experience and training in morphology. There appears to be, to the best of our knowledge, no existing published literature that evaluates the performance of the Cellavision DC-1 digital morphology analyser on chronic and acute leukaemia samples.

Automation has been implemented in the detection of leukaemia with the aim of improving early diagnosis and, subsequently, timely and effective treatment to achieve the best chance for recovery [16]. Thus, the objective of this study was to evaluate the performance of the DC-1 digital morphology analyser on the morphological classification of leukaemia. Based on the previous literature, it is hypothesised that the DC-1 will display a better performance for the classification of neutrophils, lymphocytes, and monocytes compared to eosinophils, basophils, immature granulocytes, and blast cells.

## 2. Materials and Methods

### 2.1. Peripheral Blood Smears

This study initially included 98 leukaemia peripheral blood smears, donated from various anonymous hospitals and medical laboratories in collaboration with RMIT university. During the course of this project, 10 peripheral blood smears were excluded, resulting in a final sample size of 88 chronic and acute leukaemia smears. The most common reason for exclusion was the inability of the DC-1 to complete scanning; common error messages that appeared following incomplete scanning described the smear of cells being too short or uneven, staining that is too light or dark, and uneven white cell distribution. In our study, since peripheral blood films were obtained from various laboratories that implement different standard procedures, the quality of smears was variable. Additionally, two plasma cell leukaemia cases were excluded from statistical analysis due to the overall small numbers of plasma cells. The distribution of included leukaemia samples is outlined in Table A1, with the total numbers and distribution of leukocytes outlined in Table A2. Each smear was analysed via manual microscopy by a medical laboratory scientist with considerable morphology experience, and a full blood examination report was supplied. The white blood cell differential data and provisional diagnoses from the reports were used for analysis. All slides that underwent manual classification were scanned and classified using the Cellavision DC-1 digital morphology analyser (Cellavision, Lund, Sweden).

### 2.2. Cellavision DC-1 Digital Morphology Analyser

Scanning of Romanowsky-stained peripheral blood smears by the DC-1 is achieved through an automated microscope, digital camera, and a CNN artificial intelligence that pre-classifies cells [17]. The analyser is connected to a desktop computer with the Cellavision software (version 7.1) downloaded. Images of scanned cells and pre-classification results are accessed from the computer and can be saved to a database for remote access. The DC-1 classifies leukocytes into 12 cell classes, including band neutrophils, segmented neutrophils, eosinophils, basophils, lymphocytes, monocytes, promyelocytes, myelocytes, metamyelocytes, blasts with no specific lineage, variant form lymphocytes, and plasma cells. The software also includes 5 non-white blood cell classes, including nucleated red blood cells, giant thrombocytes, thrombocyte aggregation, smudge cells, and artefacts, in addition to a miscellaneous category titled ‘other’. Before scanning of peripheral blood smears, a cell location test for peripheral blood is performed as a quality control measure. The cell location test involves the scanning of a quality control slide, the analyser will then attempt to locate 200 leukocytes, the results are then verified manually to ensure that the scanner has met satisfactory quality specifications. A drop of Cellavision immersion oil, XU-10319, is added to the reading frame of every peripheral blood slide prior to scanning.

### 2.3. Statistical Analysis: Specificity, Sensitivity, and Cohen’s Kappa

**Figure A1:**
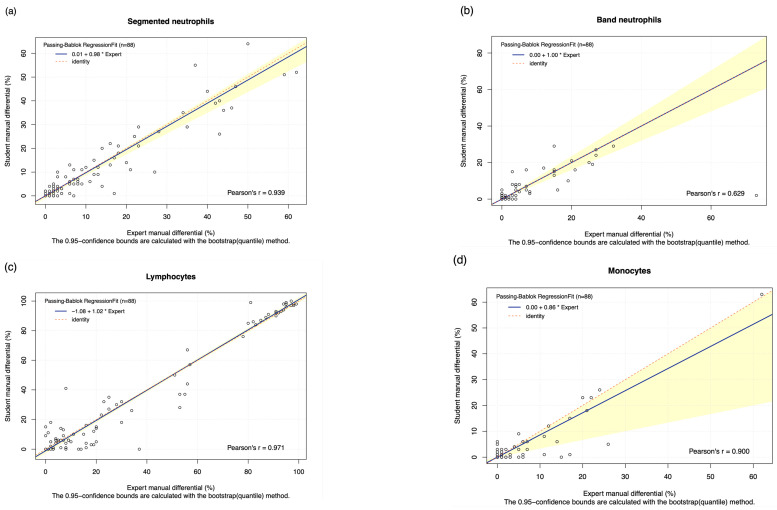

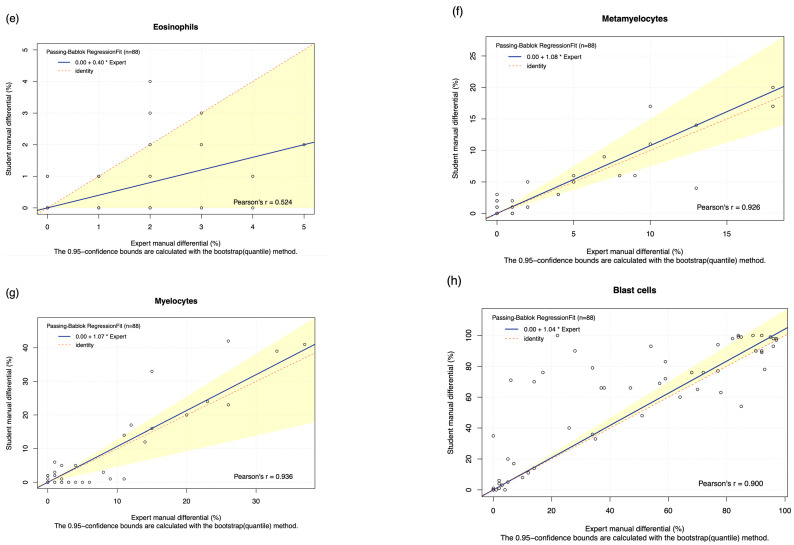
Student manual differential vs. medical scientist manual differential. The Passing–Bablok Regression fit showing the correlation and the linear relationship between the DC-1 post-classification (y-axis) and the medical scientist manual differential (x-axis). (**a**) Neutrophils, (**b**) Band neutrophils, (**c**) Lymphocytes, (**d**) Monocytes, (**e**) Eosinophils, (**f**) Metamyelocytes, (**g**) Myelocytes, and (**h**) Blast cells. The blue line represented the regression fit line, with the red dotted line showing the ideal agreement line, and the yellow shadow highlighting the 95% CI around the regression line. The Passing–Bablok regression fit for basophils and promyelocytes were not shown due to the non-linear relationship observed.

**Figure A2:**
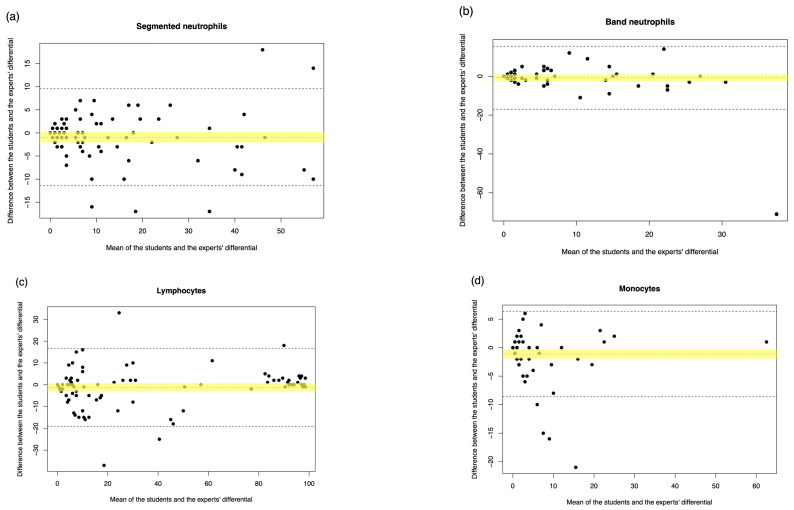

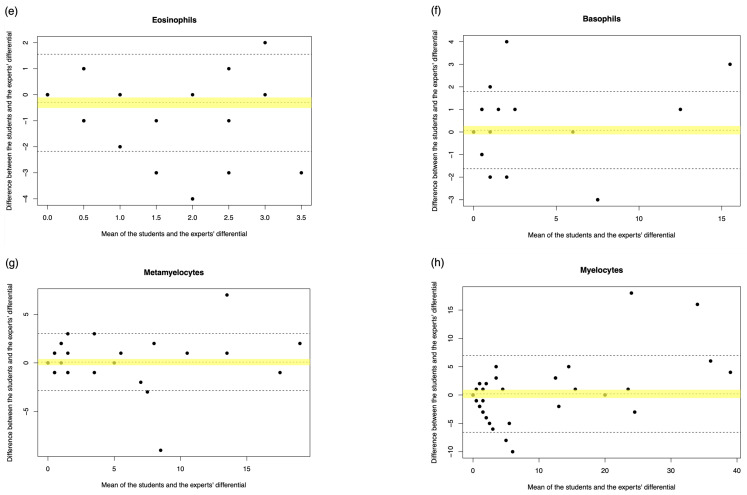

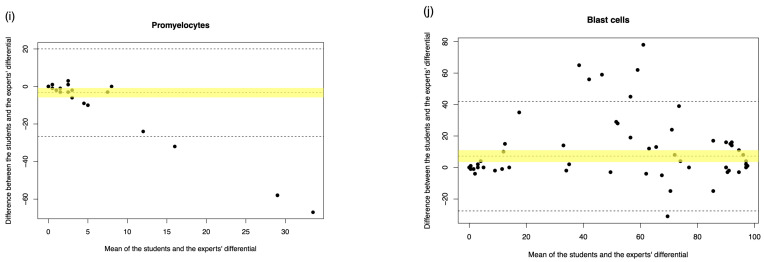
Student manual differential vs. medical scientist manual differential. The Bland–Altman plot showed the mean difference between the student and the medical scientist’s differential, with the difference (y-axis) versus the mean (x-axis). (**a**) Segmented neutrophils, (**b**) Band neutrophils, (**c**) Lymphocytes, (**d**) Monocytes, (**e**) Eosinophils, (**f**) Basophils, (**g**) Metamyelocytes, (**h**) Myelocytes, (**i**) Promyelocytes, and (**j**) Blast cells. The middle dotted line represented the mean difference, and the middle, with the yellow shadow, highlighted the 95% CI of the mean difference. The upper and lower dotted line represented the 95% CI of the limits of agreement.

Following completion of scanning and pre-classification by the DC-1, all scanned images of cells were post-classified remotely by two laboratory medicine students. In order to verify the competency of the student post-classification results, student manual microscopy was assessed in comparison with experienced medical scientist manual results, as displayed in Table A3 and Figure A1 and Figure A2. Weighted Cohen’s kappa was used to describe the overall agreement between DC-1 pre- and post-classification, in addition to pre-classification sensitivity and specificity calculations for each leukocyte class. Weighted Cohen’s kappa values are a measure of interrater reliability that range from −1 to +1, and are interpreted as none: ≤0, slight: 0.01–0.20, fair: 0.21–0.40, moderate: 0.41–0.60, substantial: 0.61–0.80, and almost perfect: 0.81–1.00 agreement [18]. Cohen’s kappa was calculated using IBM SPSS statistics 29.0 [19].

### 2.4. Statistical Analysis: Correlation, Accuracy, and Bias

The DC-1 pre-classification and post-classification results were also compared with the medical laboratory scientist manual results for the identification of segmented neutrophils, band neutrophils, lymphocytes, monocytes, eosinophils, basophils, metamyelocytes, myelocytes, promyelocytes, and blast cells. The manual microscopy results were treated as true positives and used as a reference for the accuracy of the DC-1 classification results, due to manual microscopy being considered the gold standard for morphological classification. Pearson’s correlation coefficients, defined as ‘r’, were calculated to determine the strength of correlation between the different methods of classification. The correlation coefficients were interpreted as negligible: <0.3, low: 0.31–0.5, moderate: 0.51–0.7, strong: 0.71–0.9, and very strong: >0.9 correlation [20]. Estimated accuracy was given as a percentage and calculated based on the agreement between the manual microscopy and DC-1 correlation results, using the formula

(1) $$\frac{Sum\;of\;the\;difference\;between\;“The\;method”\;and\;“The\;expert”\;results}{Sum\;of\;“The\;expert”\;and\;“The\;method”\;results\;}\times 100\%$$

Passing–Bablok regression and Bland–Altman analysis were undertaken to identify the presence of constant and proportional, as well as systematic, bias between the different classification methods. Bias was considered to be detected if the 95% CI of the mean difference and intercept did not include the value of zero, and if the 95% CI of the slope did not include the value of 1. Passing–Bablok and Bland–Altman analysis was performed using Rstudio [21], *p*-values < 0.05 were considered statistically significant.

## 3. Results

### 3.1. DC-1 Pre-Classification Performance

The DC-1 pre-classification specificity and sensitivity for each leukocyte class is outlined in Table 1. A matrix table providing a cell-by-cell comparison between DC-1 pre- and post-classification is presented in Table 1. The DC-1 was able to correctly classify 4973 leukocytes out of 7726 intact scanned leukocytes, with 4937 being the total number of true positives (Table 1), and had an overall weighted Cohen’s kappa coefficient of 0.52, a 95% confidence interval of (0.51, 0.54), and a *p*-value of <0.001. This reveals a statistically significant moderate overall agreement between pre-classification and post-classification. The specificity of the DC-1 pre-classification across all leukocyte classes was very high, with percentages above 94%. The exception was blast cells, with a high specificity of 71.3%. In contrast, the DC-1 pre-classification sensitivity for leukocytes was more varied. The DC-1 showed a high sensitivity for the pre-classification of segmented neutrophils, band neutrophils, and blast cells, with percentages between 72% and 86%, whereas a moderate sensitivity was displayed for the pre-classification of lymphocytes and metamyelocytes, with percentages of 61.4% and 59.5%, respectively, as well as low and negligible pre-classification sensitivities for monocytes, eosinophils, basophils, myelocytes, and promyelocytes, displaying percentages below 50%.

### 3.2. Comparison of Methods: DC-1 Pre-Classification

**Figure 1:**
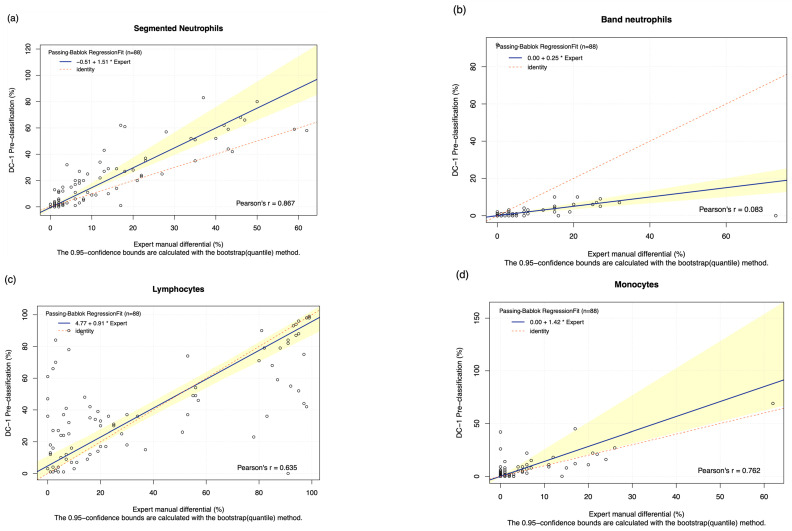

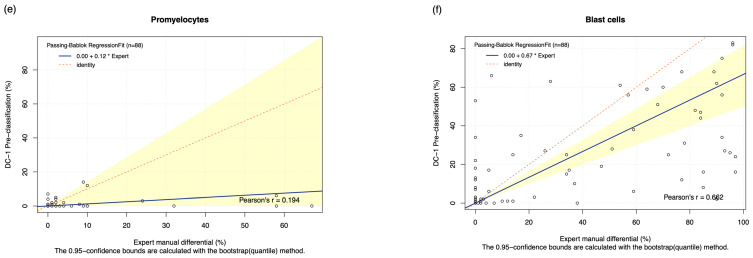
The Passing–Bablok Regression Fit showing the correlation and the linear relationship between the DC-1 pre-classification (*y*-axis) and the manual differential (*x*-axis). (**a**) Neutrophils, (**b**) Band neutrophils, (**c**) Lymphocytes, (**d**) Monocytes, (**e**) Promyelocytes and (**f**) Blast cells. The navy bold line represented the regression fit line, with the red dotted line showing the ideal agreement line, and the yellow shadow highlighting the 95% CI around the regression line. The Passing–Bablok regression fit for eosinophils, basophils, metamyelocytes, and myelocytes displayed a non-linear relationship and thus were excluded from further analysis. Overall, it showed that Cellavision DC-1 pre-classification exhibits satisfactory results for segmented neutrophils, lymphocytes, monocytes, and blast cells.

**Figure A3:**
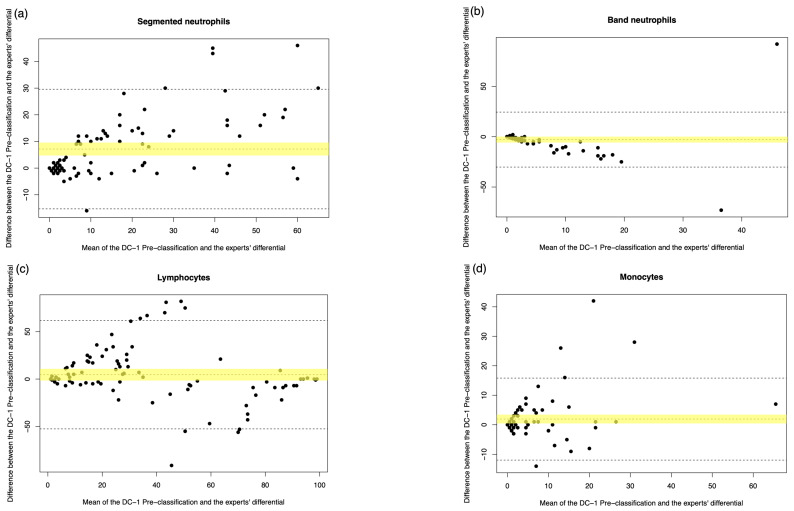

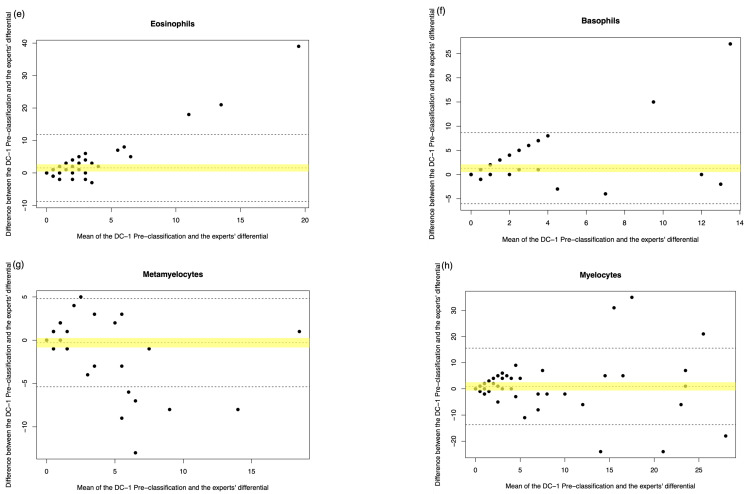

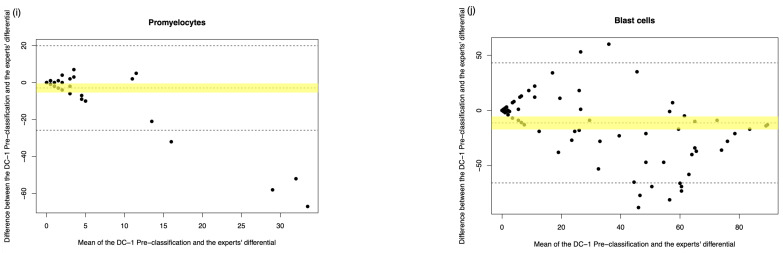
DC-1 pre-classification vs. medical scientist manual differential. The Bland–Altman plot showed the mean difference between the DC-1 pre-classification and the medical scientist’s differential, with the difference (y-axis) versus the mean (x-axis). (**a**) Segmented neutrophils, (**b**) Band neutrophils, (**c**) Lymphocytes, (**d**) Monocytes, (**e**) Eosinophils, (**f**) Basophils, (**g**) Metamyelocytes, (**h**) Myelocytes, (**i**) Promyelocytes, and (**j**) Blast cells. The middle dotted line represented the mean difference, and the middle, with the yellow shadow, highlighted the 95% CI of the mean difference. The upper and lower dotted line represented the 95% CI of the limits of agreement.

The pre-classification performance of the DC-1 was evaluated through comparison with experienced morphologist manual microscopy results using Passing–Bablok and Bland–Altman analysis, with the Bland–Altman graphs displayed in Figure A3. The results displayed a moderate to high estimated accuracy of above 60% for segmented neutrophils, lymphocytes, monocytes, and blast cells, as well as a moderate to strong correlation with the manual results for these cell classes, with Pearson’s r coefficient values of between 0.6 and 0.8 (Table 2, Figure 1a,c,d,f). A fair estimated accuracy was found for metamyelocytes and myelocytes, with an estimated accuracy of between 50 and 60%. However, low estimated accuracies and negligible correlation with the manual microscopy results were observed for band neutrophils and promyelocytes, with estimated accuracies lower than 31% and correlation coefficients lower than 0.2 (Table 2, Figure 1b,e).

The Passing–Bablok 95% confidence interval (CI) of slope did not include the value of 1 for segmented neutrophils and blast cells, suggesting the existence of proportional bias. Due to the non-linear relationship observed, no proportional and constant bias was able to be detected for eosinophils, basophils, metamyelocytes, and myelocytes. The Bland–Altman analysis of the mean difference, 95% CI, failed to capture 0 for segmented neutrophils, monocytes, eosinophils, basophils, and blasts, suggesting the presence of systematic bias (Table 2).

### 3.3. Comparison of Methods: DC-1 Post-Classification

**Figure 2:**
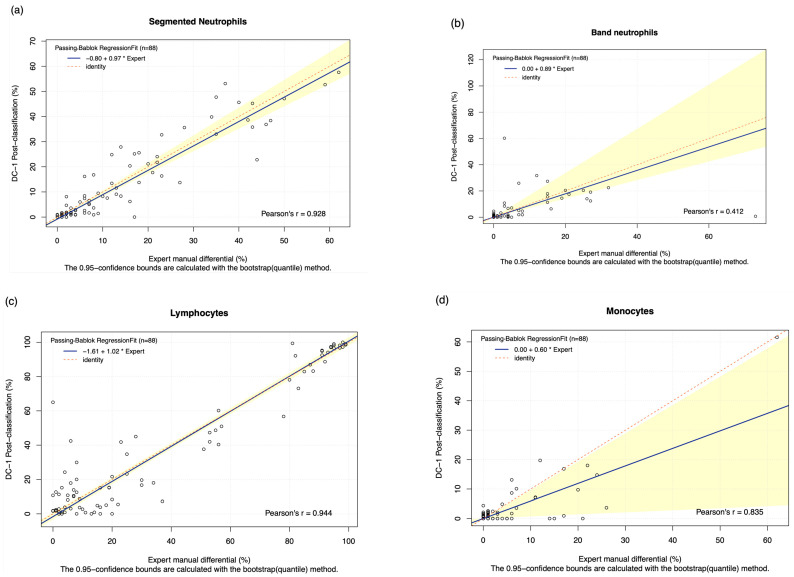

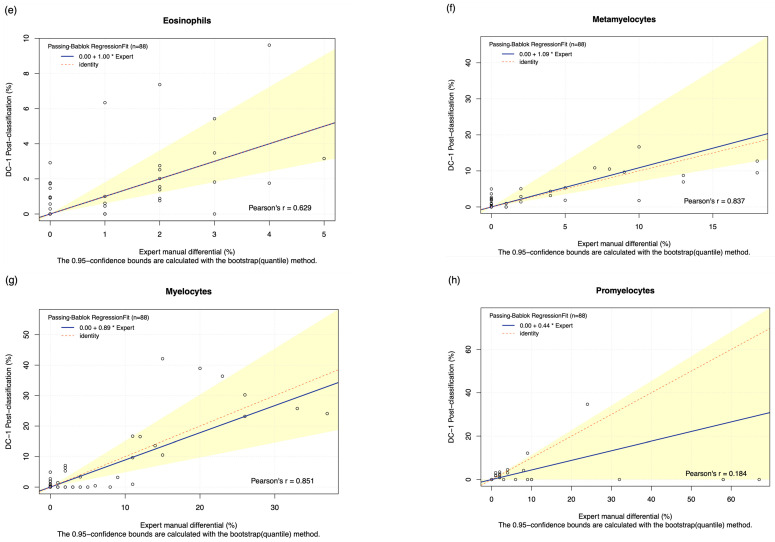

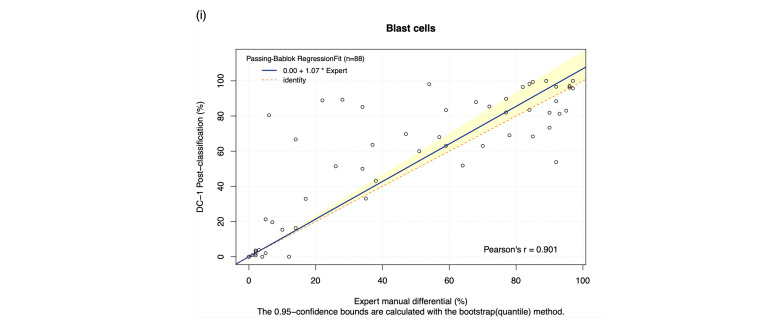
The Passing–Bablok Regression fit showing the correlation and the linear relationship between the DC-1 post-classification (*y*-axis) and the manual differential (*x*-axis). (**a**) Neutrophils, (**b**) Band neutrophils, (**c**) Lymphocytes, (**d**) Monocytes, (**e**) Eosinophils, (**f**) Metamyelocytes, (**g**) Myelocytes, (**h**) Promyelocytes, and (**i**) Blast cells. The blue line represented the regression fit line, with the red dotted line showing the ideal agreement line, and the yellow shadow highlighting the 95% CI around the regression line. The Passing–Bablok regression fit for basophils displayed a non-linear relationship and thus were excluded from further analysis. Overall, it showed that Cellavision DC-1 post-classification exhibits satisfactory performance for segmented neutrophils, lymphocytes, monocytes, metamyelocytes, myelocytes, and blast cells.

**Figure A4:**
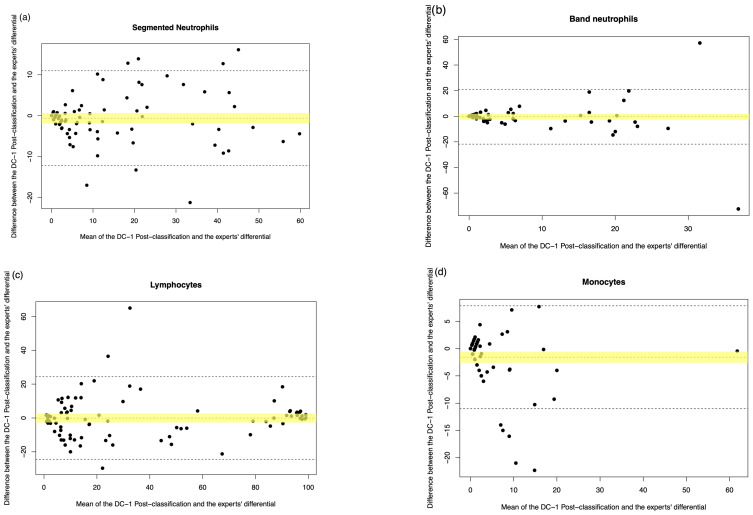

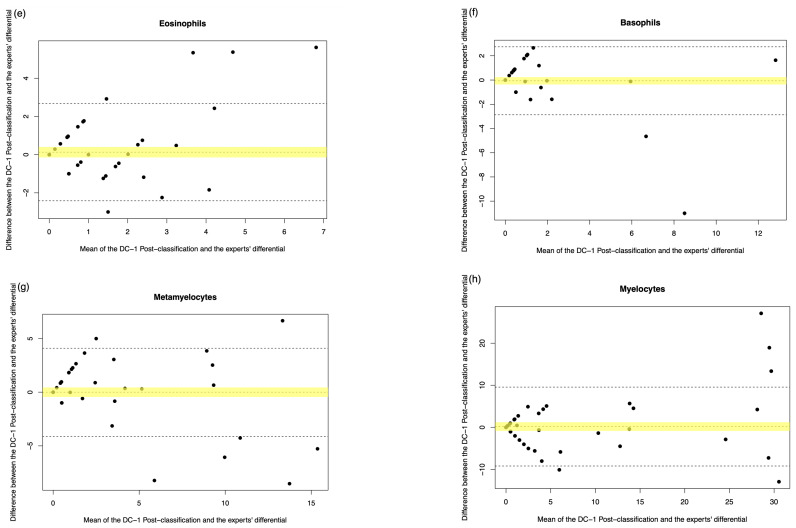

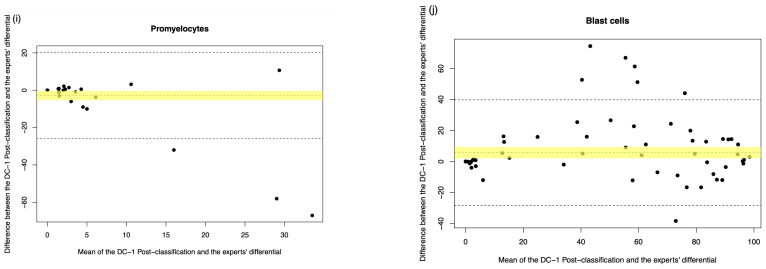
DC-1 post-classification vs. medical scientist manual differential. The Bland–Altman plot showed the mean difference between the DC-1 post-classification and the medical scientist’s differential, with the difference (y-axis) versus the mean (x-axis). (**a**) Segmented neutrophils, (**b**) Band neutrophils, (**c**) Lymphocytes, (**d**) Monocytes, (**e**) Eosinophils, (**f**) Basophils, (**g**) Metamyelocytes, (**h**) Myelocytes, (**i**) Promyelocytes, and (**j**) Blast cells. The middle dotted line represented the mean difference, and the middle, with the yellow shadow, highlighted the 95% CI of the mean difference. The upper and lower dotted line represented the 95% CI of the limits of agreement.

The DC-1 scans of leukaemia peripheral blood smears were manually post-classified and compared with experienced morphologist manual microscopy results using Passing–Bablok and Bland–Altman analysis, with the Bland–Altman graphs displayed in Figure A4. A high estimated accuracy of above 80% was observed for neutrophils, lymphocytes, and blast cells, as well as a very strong correlation with the manual microscopy results for these cell classes, indicated by Pearson’s r coefficient values of greater than 0.9 (Table 2, Figure 2a,c,i). Band neutrophils, monocytes, eosinophils, basophils, metamyelocytes, and myelocytes showed a moderate estimated accuracy of between 54 and 72%. Monocytes, metamyelocytes, and myelocytes displayed a strong correlation strength with the manual microscopy results, with Pearson’s r coefficient values of greater than 0.8. Eosinophils displayed moderate correlation, with an r coefficient of 0.629, whereas band neutrophils showed a relatively weak correlation strength with the manual microscopy results, with a Pearson’s r coefficient value of 0.412 (Table 2, Figure 2b,d,f,g). A low estimated accuracy of less than 30% and a weak correlation strength were observed for promyelocytes, with a Pearson’s r coefficient value of 0.184 (Table 2, Figure 2e). The 95% CI of intercept failed to capture 0 for segmented neutrophils, suggesting the existence of constant bias. The Bland–Altman results revealed that the 95% CI of the mean difference failed to capture 0 for neutrophils, monocytes, promyelocytes, and blast cells, suggesting the existence of systematic bias. A non-linear relationship was observed for basophils, resulting in a failure to detect proportional and constant bias provided by Passing–Bablok analysis.

## 4. Discussion

The aim of this study was to evaluate the performance of the DC-1 on leukaemia samples, determining if it is suitable for the identification of leukaemia in low-throughput or remote laboratories.

The pre-classification performance of the DC-1 displayed a moderate to strong estimated accuracy and correlation with manual results for segmented neutrophils, lympho cytes, and blast cells; this is displayed in Figure 1a,c,f, and Table 2. A fair pre-classification performance was shown for metamyelocytes and myelocytes, with a negligible performance for band neutrophils and promyelocytes. The positive proportional and systemic bias detected for segmented neutrophils indicates that the segmented neutrophil count was likely overestimated by the DC-1 pre-classification, as presented in Table 2 and Table 3. In order to comply with ISO 15189 and 17025 [22], all medical laboratory results must be verified by a medical laboratory scientist to ensure quality patient safety. The bias displayed by the DC-1 for the pre-classification of neutrophils was correct upon post-classification, suggesting that the performance is within an acceptable margin of error and is not likely to negatively impact clinical decision making on leukaemia. Previous studies on the DC-1 have focused on normal peripheral blood smears, or samples with slightly elevated or reduced leukocyte counts, showing that the DC-1 is more precise in the classification of smears with normal cell counts. The DC-1 has also shown a relatively high correlation with manual microscopy and the Sysmex DI-60 digital morphology analyser, for the classification of normal segmented neutrophils, lymphocytes, and monocytes [1,14]. In contrast, our study focuses solely on leukaemia samples that tend to display very high leukocyte counts. Our results, compared with previous studies on the DC-1, indicate that with increasing leukocyte numbers, the DC-1 shows a decreasing trend in accuracy. This suggests that the DC-1 may be best suited for small to medium workload laboratories that receive samples with either normal or slightly increased or decreased leukocyte counts. Studies evaluating the pre-classification performance of other digital morphology analyser models, such as the Cellavision DM-series, DI-60, and Mindray MC series, were fairly similar to the results of this study and revealed a good correlation between automated and manual classification for most cell types, except for basophils, myelocytes, metamyelocytes, and band neutrophils [4,6,10,23,24]. These digital analyser models have a higher throughput and are more suited to medical laboratories with a heavy workload; in comparison the DC-1 is more cost-effective for low throughput laboratories, for instance, regional core laboratories with limited budgets or insufficient manpower. The use of the Cellavision DC-1 digital morphology analyser could possibly support remote morphological classification in laboratories that require support from off-site morphologists in main laboratories.

The less than satisfactory DC-1 pre-classification results for immature granulocytes and basophils, as well as the high variability seen in blast cell classification, as seen in Table 2 and Table 3, reveals that the DC-1 pre-classification may not be adequate for the morphological diagnosis of CL and AL with large numbers of immature granulocytes and blast cells. The diagnosis of leukaemia can be very complex and often requires a number of techniques, including morphology, cytogenetics, immunophenotyping, clinical findings, and cytochemistry, as opposed to one standalone technique [25]. Therefore, despite our study revealing that the DC-1 displayed a poorer performance in the differentiation of immature granulocytes, basophils, and blast cells compared to other cell types, the DC-1’s ability of flag the presence of potential leukaemic cells makes it a useful tool to identify complex samples that require further investigation. It is recommended by the ICSH guidelines that manual verification using light microscopy be used when suspecting leukaemia or the presence of blast cells [26], therefore the DC-1 could benefit diagnostic workflow in small to moderate workload laboratories by aiding morphologists in deciding how to use their time effectively and which smears require expert morphological review. With the development of AI neural networks and their implementation in laboratory medicine, there is a possibility for improved performance of digital morphology analysers in the differentiation of different cell types.

Other digital morphology analyser models, especially the Mindray MC-series, have shown better accuracy in the pre-classification of blast cells and other pathological cells, compared to the DC-1 [11,27]. The Cellavision DM-series, particularly Cellavision DM-96, has shown a promising diagnostic ability for the detection of blast cells [28] as well as high correlation and accuracy for the pre-classification of leukocytes in samples with qualitative and quantitative leukocyte abnormalities [4]. A recent study evaluated the ability of Cellavision to replace manual classification and verification of segmented neutrophils and revealed consistently high correlations between pre- and post-classification and satisfactory diagnostic performance [8]. Our results also show very high specificity, sensitivity, and correlation for the pre-classification of segmented neutrophils by the Cellavision DC-1 found in Table 1 and Table 2, which suggests the possible use of the DC-1 to aid in the diagnosis of patients with bacterial infection or neutropenic fever [8].

An overall improvement was seen in the DC-1 post-classification results compared to pre-classification results particularly for immature granulocytes and blast cells; this is seen in Figure 2f–i, and Table 2 and Table 3. Suggesting that post-classification is required for more reliable results and pre-classification alone is insufficient, especially when differentiating between CL and AL. DC-1 post-classification showed a strong estimated accuracy and correlation for segmented neutrophils, lymphocytes, blast cells, monocytes, metamyelocytes, and myelocytes. In terms of monocytes, the DC-1 showed a moderate correlation with the manual results, with a similar performance for both pre- and post-classification. It is important to note that there was a weak post-classification accuracy and correlation for basophils and promyelocytes, which are important features to identify basophilia in CML, or increased numbers of promyelocytes in APML. The moderate post-classification estimated accuracy and correlation coefficients for band neutrophils presented in Table 2 and Table 3 may partly be due to interobserver variation that has been commonly reported in the classification of this cell type [29,30,31]. Overall, results from previous studies concerning the post-classification performance of different digital morphology analyser models have shown strong accuracy and correlations with manual microscopy for segmented neutrophils and lymphocytes, a lower accuracy and correlation for basophils and band neutrophils, and varying results for monocytes and eosinophils [2,10,23].

The main limitation of this study would be the staining and smear quality of the leukaemia samples. Staining that is too light or dark, as well as a smear that is uneven or too short, can affect the ability of the DC-1 to complete scanning and correctly identify leukocytes. However, leukaemia samples with high leukocyte counts, dark granules, and accumulation of proteins can make it difficult to achieve high-quality stain and smear preparation. Cellavision has recommended instructions for the preparation of peripheral blood slides, including the length of the smear and staining method. Laboratories that decide to introduce the DC-1 digital morphology analyser are suggested to follow the recommended instructions from Cellavision for the standardisation of slide preparation. Additionally, a semi-automated slide-maker from Cellavision, such as the Cellavision HemaPrep [32], could be introduced to the laboratory to improve standardisation of slide quality. In our study, peripheral blood films were obtained from various laboratories that used different operating procedures; the slides of poorer quality that caused incomplete scanning and were flagged by the DC-1 had to be excluded. Therefore, adhering to the slide preparation instructions from Cellavision should be considered to possibly significantly reduce scanning failure in a real-world laboratory setting.

## 5. Conclusions

This study is the first to analyse the performance of the DC-1 digital morphology analyser on leukaemia samples. The DC-1 displayed a strong performance for the pre- and post-classification of segmented neutrophils and lymphocytes, particularly in the identification of neutrophilia and lymphocytosis. However, it was weak in identifying immature granulocytes, eosinophils, and basophils, especially promyelocytes, indicating that the DC-1 may be more suitable for use on peripheral blood smears that are normal or displaying bacterial or viral infection, as opposed to leukaemia samples with high numbers of immature granulocytes, blasts, and dysplastic cells. Post-classification is able to provide more accurate results than pre-classification, however manual light microscopy still remains the gold standard for the morphological classification of leukaemia cases. The DC-1 may also be used to flag the presence of immature granulocytes and blasts in complex samples for further investigation and may be a beneficial addition to remote or low-volume laboratories that require support from off-site morphologists in main laboratories.

## Figures and Tables

**Table 1:** Cellavision DC-1 pre-classification versus post-classification matrix table depicting sensitivity and specificity of leukocyte pre-classification from leukaemia peripheral blood smears ^1^.

*Pre-Classification*											
Post- Classification	Segmented Neutrophils	Band Neutrophils	Lymphocytes	Monocytes	Eosinophils	Basophils	Metamyelocytes	Myelocytes	Promyelocytes	Blasts	Post- Classification Total
Segmented Neutrophils	**997**	4	3	2	3	5	0	1	0	0	1015
Band neutrophils	209	**70**	5	0	6	0	0	1	0	0	291
Lymphocytes	46	0	**2101**	43	1	8	4	44	0	180	2427
Monocytes	8	0	3	**189**	0	0	0	0	0	0	200
Eosinophils	2	1	0	0	**55**	2	0	2	0	0	62
Basophils	0	0	10	0	0	**42**	0	2	1	1	56
Metamyelocytes	45	6	16	5	1	0	**50**	2	0	1	126
Myelocytes	17	0	137	7	3	2	5	**79**	0	1	251
Promyelocytes	22	0	4	6	2	12	0	24	**15**	33	98
Blasts	53	0	1142	279	81	88	25	103	54	**1375**	3200
Pre- classification Total	1379	81	3421	531	152	159	84	258	70	1591	**7726**
True Positive	997	70	2101	189	55	42	50	79	15	1375	-
False Negative	382	11	1320	342	97	117	34	179	55	216	-
True Negative	6711	7435	5299	7526	7664	7670	7600	7475	7628	4526	-
False Positive	18	221	326	11	7	14	76	172	83	1825	-
Specificity (%)	99.7	97.1	94.2	99.9	99.9	99.8	99	97.8	98.9	71.3	-
Sensitivity (%)	72.3	86.4	61.4	35.6	36.2	26.4	59.5	30.6	21.4	86.4	-

^1^ This matrix table displays how the specificity and sensitivity of DC-1 pre-classification of each leukocyte class was calculated. Bolded numbers represent the cells classified identically by pre- and post-classification, or the true positive (TP) values. The false negative (FN) values were calculated by subtracting the TP values from the pre-classification total for each cell type. The true negative (TN) values were calculated by subtracting the post-classification total for each cell type from the absolute total number of cells. The false positive (FP) values were calculated by subtracting the TP values from the post-classification total for each cell type. Specificity was calculated by dividing the TN values by the sum of the TN and FP values, while sensitivity was calculated by dividing the TP values by the sum of the TP and FN values.

**Table 2:** The estimated accuracy, Pearson’s (r) coefficient values, Passing–Bablok regression, and Bland–Altman analysis on the DC-1 pre-classification and post-classification results, in comparison with the manual microscopy results, for leukaemia samples ^1^.

Leukocytes	Pre-Classification					Post-Classification				
	Estimated Accuracy	r (95% CI)	Slope (95% CI)	Intercept (95% CI)	Mean Difference (95% CI)	Estimated Accuracy	r (95% CI)	Slope (95% CI)	Intercept (95% CI)	Mean Difference (95% CI)
Segmented neutrophils	74.757%	0.867 (*p* < 0.001)	1.511 (1.353 to 1.868)	−0.511 (−1.568 to 0.711)	7.148 (4.721 to 9.574)	84.823%	0.928 (*p* < 0.001)	0.970 (0.897 to 1.117)	−0.799 (−1.147 to −0.037)	−0.613 (−1.865 to 0.640)
Band neutrophils	30.341%	0.083 (*p* = 0.444)	0.250 (0.167 to 0.333)	0.000 (0.000 to 0.000)	−2.864 (−5.815 to 0.088)	60.697%	0.412 (*p* < 0.001)	0.892 (0.707 to 1.683)	0.000 (0.000 to 0.000)	0.355 (−2.669 to 1.958)
Lymphocytes	74.786%	0.635 (*p* < 0.001)	0.909 (0.800 to 1.000)	4.773 (0.000 to 10.600)	4.568 (−1.610 to 10.747)	88.786%	0.944 (*p* < 0.001)	1.023 (0.990 to 1.054)	−1.614 (−3.460 to 0.808)	−0.046 (−2.683 to 2.591)
Monocytes	65.297%	0.762 (*p* < 0.001)	1.417 (1.000 to 2.591)	0.000 (−0.265 to 0.400)	1.943 (0.443 to 3.443)	63.10%	0.835 (*p* < 0.001)	0.595 (0.058 to 0.961)	0.000 (0.000 to 0.000)	−1.573 (−2.593 to −0.554)
Eosinophils	29.508%	* 0.448 (*p* < 0.001)	N.A.	N.A.	1.545 (0.428 to 2.663)	54.226%	0.629 (*p* < 0.001)	1.000 (0.606 to 2.020)	0.000 (0.000 to 0.000)	0.129 (−0.146 to 0.404)
Basophils	38.222%	* 0.336. (*p* = 0.001)	N.A.	N.A.	1.307 (0.514 to 2.100)	61.67%	* 0.567 (*p* < 0.001)	N.A.	N.A.	−0.062 (−0.366 to 0.241)
Metamyelocytes	56.911%	* 0.592 (*p* < 0.001)	N.A.	N.A.	−0.295 (−0.846 to 0.256)	69.203%	0.837 (*p* < 0.001)	1.087 (0.707 to 2.944)	0.000 (0.000 to 0.000)	−0.002 (−0.446 to 0.441)
Myelocytes	55.241%	* 0.542 (*p* < 0.001)	N.A.	N.A.	0.932 (−0.652 to 2.515)	71.297%	0.851 (*p* < 0.001)	0.891 (0.438 to 1.515)	0.000 (0.000 to 0.000)	0.198 (−0.814 to 1.211)
Promyelocytes	19.90%	0.194 (*p* = 0.070)	0.125 (0.000 to 1.200)	0.000 (0.000 to 0.000)	−2.932 (−5.397 to −0.466)	27.56%	0.184 (*p* = 0.09)	0.442 (0.000 to 1.136)	0.000 (0.000 to 0.000)	−2.836 (−5.534 to −0.337)
Blast cells	62.920%	0.662 (*p* < 0.001)	0.667 (0.500 to 0.815)	0.000 (0.000 to 0.261)	−11.341 (−17.218 to − 5.464)	86.193%	0.901 (*p* < 0.001)	1.069 (0.994 to 1.170)	0.000 (0.000 to 0.000)	5.744 (2.064 to 9.424)

^1^ The degree of correlation between DC-1 performance (pre-classification and post-classification) and manual microscopy is described by Pearson’s correlation coefficients for each leukocyte class, as well as presented as a percentage by the estimated accuracy. Pearson’s r results displayed a strong correlation between DC-1 pre-classification and manual microscopy for segmented neutrophils and monocytes, while a moderate correlation was displayed for lymphocytes, metamyelocytes, myelocytes, and blast cells, a weak or negligible correlation for band neutrophils, eosinophils, basophils, and promyelocytes. For DC-1 post-classification correlation with manual microscopy, Pearson’s r results displayed a strong correlation for segmented neutrophils, lymphocytes, monocytes, metamyelocytes, myelocytes, and blasts, as well as a moderate correlation for eosinophils and basophils, while a weak or negligible correlation was shown for band neutrophils and promyelocytes. The 95% CI of the slope and the intercept for different cells were measured by Passing–Bablok regression, and the 95% CI of the mean difference for different cells were measured by Bland–Altman analysis; this was used to identify the presence of bias. * For the DC-1 pre-classification, the Pearson’s correlation was not applicable on eosinophils, basophils, metamyelocytes, and myelocytes, due to non-linear relationships observed, and correlation was determined by Spearman’s rank-order correlation, with Spearman’s ρ value of 0.448 (*p* < 0.001), 0.336 (*p* = 0.001), 0.592 (*p* < 0.001), and 0.542 (*p* < 0.001), respectively. * For the DC-1 post-classification, the Pearson’s correlation was not applicable on basophils, due to the non-linear relationships observed, and correlation was determined by Spearman’s rank-order correlation, with Spearman’s ρ value of 0.567 (*p* < 0.001). N.A. refers to “not available”, meaning that the slope and intercept were unable to be determined due to the non-linear relationships observed, where proportional and constant bias could not be detected by Passing-Bablok regression.

**Table 3:** Summary table of the accuracy, correlation, and bias results for the analysis of DC-1 pre- and post-classification of leukaemia peripheral blood smears, in comparison with manual microscopy results.

Accuracy and Correlation (with Bias Analysis)		
	DC-1 Pre-Classification	DC-1 Post-Classification
Segmented neutrophils	High estimated accuracy and strong correlation strength with the manual microscopy results. Overestimated count shown by the positive constant and proportional bias.	High estimated accuracy and strong correlation strength with the manual microscopy results. No bias detected.
Band neutrophils	Low estimated accuracy and negligible correlation strength with the manual microscopy results. No bias detected.	Moderate estimated accuracy and relatively weak correlation strength with the manual microscopy results. No bias detected.
Lymphocytes	High estimated accuracy and moderate correlation strength with the manual microscopy results. No bias detected.	High estimated accuracy and strong correlation strength with the manual microscopy results. No bias detected.
Monocytes	Moderate estimated accuracy with a strong correlation strength with the manual microscopy results. Mild positive systemic bias detected.	Moderate estimated accuracy with strong correlation strength with the manual microscopy results. Mild negative proportional bias detected.
Eosinophils	Low estimated accuracy with a weak correlation strength with the manual microscopy results. Proportional and constant bias failed to be detected due to the non-linear relationship observed. Mild positive systemic bias detected. Low sample size reducing the statistical significance.	Moderate estimated accuracy with a moderate correlation strength with the manual microscopy results. No bias detected. Low sample size reducing the statistical significance.
Basophils	Low estimated accuracy with a weak correlation strength with the manual microscopy results. Proportional and constant bias failed to be detected due to the non-linear relationship observed. Mild positive systemic bias detected. Low sample size reducing the statistical significance.	Moderate estimated accuracy and moderate correlation strength with the manual microscopy results. Proportional and constant bias failed to be detected due to the non-linear relationship observed. Low sample size reducing the statistical significance.
Metamyelocytes	Moderate estimated accuracy with a moderate correlation strength with the manual microscopy results. Proportional and constant bias failed to be detected due to the non-linear relationship observed. Low sample size reducing the statistical significance.	Moderate estimated accuracy with a strong correlation strength with the manual microscopy results. No bias detected. Low sample size reducing the statistical significance.
Myelocytes	Moderate estimated accuracy with a moderate correlation strength with the manual microscopy results. Proportional and constant bias failed to be detected due to the non-linear relationship observed.	High estimated accuracy with a strong correlation strength with the manual microscopy results.
Promyelocytes	Extremely low estimated accuracy with a weak correlation strength with the manual microscopy results. Mild negative systemic bias detected.	Low estimated accuracy with a very weak correlation strength with the manual microscopy results. Mild negative systemic bias detected.
Blast cells	Moderate estimated accuracy and correlation strength with the manual microscopy results. High variability shown by Passing–Bablok Regression Fit. Negative proportional and systemic bias detected.	High estimated accuracy and strong correlation strength with the manual microscopy results. Positive systemic bias detected in this study.

## Data Availability

During data collection and analysis phases, all data were securely stored at RMIT University in compliance with relevant data protection regulations. Data will only be accessible to the research team. Retention Period: Data will be retained for the duration of the project (up to 3 years) and will be disposed of in ac-cordance with data retention policies and regulations once the project is complete.

## Abbreviations

The following abbreviations are used in this manuscript:

CNN	Convolutional Neural Network
ANN	Artificial Neural Network
RMIT	Royal Melbourne Institute of Technology
CML	Chronic Myeloid Leukaemia
CLL	Chronic Lymphocytic Leukaemia
JMML	Juvenile Myelomonocytic Leukaemia
PLL	Prolymphocytic Leukaemia
HCL	Hairy Cell Leukaemia
CMML	Chronic Myelomonocytic Leukaemia
ALL	Acute Lymphoblastic Leukaemia
AML	Acute Myeloid Leukaemia
APML	Acute Promyelocytic Leukaemia
AMML	Acute Myelomonocytic Leukaemia
AMoL	Acute Monoblastic/cytic Leukaemia

## Appendix A

**Table A1:** The frequency and proportion of leukaemia samples scanned by the DC-1 ^1^.

Leukaemia Cases	Sample Size (n = 88)	Proportion (%) in Total
CML	15	16.3
CLL	18	20.7
JMML	1	1.1
PLL	6	6.5
HCL	2	3.3
CMML	3	5.4
ALL	18	19.6
AML M0	1	1.1
AML M1	4	4.3
AML M2	3	3.3
APML M3	5	5.4
AMML M4	4	4.3
AMoL M5	2	2.2
AML M6	2	2.2
AML M7	2	2.2
AML recurrent	2	2.2

^1^ CML = Chronic Myelocytic Leukaemia; CLL = Chronic Lymphocytic Leukaemia); JMML = Juvenile Myelomonocytic Leukaemia; PLL = Prolymphocytic Leukaemia; HCL = Hairy Cell Leukaemia; CMML = Chronic Myelomonocytic Leukaemia; ALL = Acute Lymphoblastic Leukaemia; AML M0 = Acute Myeloid Leukaemia minimally differentiated; AML M1 = Acute Myeloid Leukaemia without maturation; AML M2 = Acute Myelocytic Leukaemia with maturation; APML M3 = Acute Promyelocytic Leukaemia; AMML M4 = Acute Myelomonocytic Leukaemia; AMoL M5 = Acute Monoblastic/cytic Leukaemia; AML M6 = Acute Erythroid Leukaemia; AML M7 = Acute Megakaryoblastic Leukaemia; AML recurrent = Acute Myeloid Leukaemia with recurrent cytogenetic abnormalities.

**Table A2:** The frequency and proportion of leukocytes identified manually from the leukaemia samples ^1^.

Leukocytes	Total Cell Number Taken for Account (n = 8799)	Proportion (%) in Total
Segmented Neutrophils	1177	13.4
Band neutrophils	449	5.1
Lymphocytes	3182	36.2
Monocytes	377	4.3
Eosinophils	54	0.6
Basophils	55	0.6
Metamyelocytes	136	1.5
Myelocytes	312	3.5
Promyelocytes	325	3.7
Blast cells	2732	31.0

^1^ The leukocyte classes highlighted in red display a low sample size, reducing the statistical significance of the analysis of these cell classes.

## Appendix B

**Table A3:** The estimated accuracy, Pearson’s (r) coefficient value, Passing–Bablok regression, and Bland–Altman analysis comparing the student manual differential results and medical scientist manual microscopy results in leukaemia samples obtained. The degree of correlation between DC-1 pre-classification and manual microscopy is described by Pearson’s correlation coefficients for each leukocyte class, as well as presented as a percentage by the estimated accuracy. The 95% CI of the slope and the intercept for different cells were measured by Passing–Bablok regression, and the 95% CI of the mean difference for different cells was measured by Bland–Altman analysis.

Leukocytes	Estimated Accuracy	Pearson’s r Coefficient Value (95% CI)	Slope (95% CI)	Intercept (95% CI)	Mean Difference (95% CI)
Neutrophils	86.268%	0.939 (*p* < 0.001)	0.975 (0.875 to 1.032)	0.013 (−1.000 to 0.133)	−0.932 (−2.065 to 0.202)
Band neutrophils	73.165%	0.629 (*p* < 0.001)	1.000 (0.800 to 1.200)	0.000 (0.000 to 0.000)	−2.515 (−0.761 to 0.992)
Lymphocytes	91.813%	0.971 (*p* < 0.001)	1.021 (1.000 to 1.038)	−1.083 (−2.591 to 0.000)	−1.25 (−3.184 to 0.684)
Monocytes	74.504%	0.900 (*p* < 0.001)	0.857 (0.332 to 1.000)	0.000 (0.000 to 0.000)	−1.125 (−1.937 to −0.313)
Eosinophils	49.383%	0.524 (*p* < 0.001)	0.400 (0.000 to 1.000)	0.000 (0.000 to 0.000)	−0.307 (−0.508 to −0.105)
Basophils	73.504%	* 0.542 (*p* < 0.001)	N.A.	N.A.	0.080 (−0.106 to 0.265)
Metamyelocytes	81.004%	0.926 (*p* < 0.001)	1.077 (0.750 to 1.500)	0.000 (0.000 to 0.000)	0.080 (−0.239 to 0.398)
Myelocytes	79.251%	0.936 (*p* < 0.001)	1.067 (0.375 to 1.273)	0.00 (0.000 to 0.000)	0.193 (−0.538 to 0.924)
Promyelocytes	13.370%	* 0.417 (*p* < 0.001)	N.A.	N.A.	−3.307 (−5.830 to −0.784)
Blast cells	86.539%	0.900 (*p* < 0.001)	1.042 (1.000 to 1.159)	0.000 (0.000 to 0.000)	7.216 (3.456 to 10.976)

* Pearson’s correlation was not applicable on basophils and promyelocytes, due to the non-linear relationships observed, and correlation was determined by Spearman’s rank-order correlation, with Spearman’s ρ value of 0.542 (*p* < 0.001) and 0.417 (*p* < 0.001), respectively. N.A. refers to “not available”, meaning that the slope and intercept were unable to be determined due to the non-linear relationships observed, where proportional and constant bias could not be detected by Passing-Bablok regression.
